# Heart Rate Recovery Index as a Functional Marker in Heart Failure with Preserved Ejection Fraction: Associations with H_2_FPEF Score, Longitudinal Systolic Function and Left Atrial Remodelling

**DOI:** 10.3390/jcm15176510

**Published:** 2026-08-23

**Authors:** Andreea Dache, Cristina Văcărescu, Minodora Teodoru, Mihai Octavian Negrea, Cristina Tudoran, Alexandra-Iulia Lazăr-Höcher, Liviu Cirin, Adelina Andreea Faur-Grigori, Bogdan-Simion Suciu, Dragoș Cozma

**Affiliations:** 1 Institute of Cardiovascular Diseases, 300310 Timisoara, Romania; andreea.cozgarea@umft.ro (A.D.); andreea.faur@umft.ro (A.A.F.-G.); bogdan.suciu@umft.ro (B.-S.S.); dragos.cozma@umft.ro (D.C.); 2 Doctoral School, “Victor Babes” University of Medicine and Pharmacy, 300041 Timisoara, Romania; alexandra.hocher@umft.ro (A.-I.L.-H.); liviu.cirin@umft.ro (L.C.); 3 County Clinical Emergency Hospital of Sibiu, 550245 Sibiu, Romania; 4 Department of Cardiology, “Victor Babes” University of Medicine and Pharmacy, 300041 Timisoara, Romania; 5 Research Center of the Institute of Cardiovascular Diseases, 300310 Timisoara, Romania; 6 Medical Clinical Department, Faculty of Medicine, “Lucian Blaga” University, 550024 Sibiu, Romania; mihaioctavian.negrea@ulbsibiu.ro; 7 Sibiu Municipal Hospital, 550169 Sibiu, Romania; 8 Department VII, Internal Medicine II, Discipline of Cardiology, “Victor Babeș” University of Medicine and Pharmacy, 300041 Timisoara, Romania; cristina.tudoran@umft.ro; 9 Department of Cardiology, County Emergency Hospital “Pius Brînzeu”, 300723 Timisoara, Romania; 10 Centre of Molecular Research in Nephrology and Vascular Disease, “Victor Babeș” University of Medicine and Pharmacy, 300041 Timisoara, Romania

**Keywords:** heart rate recovery index, H_2_FPEF score, systolic longitudinal dysfunction, left atrial remodelling, chronic heart failure, management of heart failure

## Abstract

**Background:** The Heart Rate Recovery Index (HRRI), derived from post-exercise heart rate recovery (HRR), reflects autonomic function and cardiovascular performance. Whether HRRI reflects early myocardial dysfunction and left atrial remodelling in heart failure with preserved ejection fraction (HFpEF) has not been previously examined. The H_2_FPEF score, which integrates clinical and echocardiographic parameters, is used to assess the likelihood of HFpEF. This study investigates the relationship between HRRI, H_2_FPEF score, and echocardiographic markers of longitudinal systolic function, including mitral annular plane systolic excursion (MAPSE), as well as left atrial volume index (LAVI), in patients with preserved left ventricular ejection fraction. **Methods:** A prospective observational study included 241 patients referred for cardiac exercise testing at the Institute of Cardiovascular Diseases Timisoara and the Clinical County Hospital of Sibiu. HRRI was calculated as the ratio of heart rate acceleration time (AT) to deceleration time (DT) during exercise testing. A comprehensive echocardiographic assessment was performed on all patients. Statistical analysis involved univariate testing and multivariable logistic regression with stepwise selection. **Results:** HRRI was significantly lower in HFpEF patients compared with those without heart failure (1.97 ± 0.66 vs. 2.73 ± 1.08, *p* < 0.01). HRRI correlated significantly with exercise performance, age, H_2_FPEF score, and echocardiographic markers of diastolic and longitudinal systolic dysfunction. ROC analysis identified an HRRI cut-off value of 2.25 for HFpEF detection (AUC = 0.748), while HRRI remained significantly associated with HFpEF after adjustment for the covariates included in the model. The combined HRRI–H_2_FPEF score improved diagnostic discrimination compared with the H_2_FPEF score alone (AUC 0.897 vs. 0.858), achieving an overall classification accuracy of 82.2%. **Conclusions:** In our study, HRRI is significantly reduced in HFpEF and distinguishes patients with and without heart failure. It shows associations with echocardiographic markers of diastolic and longitudinal systolic dysfunction, exercise capacity, and H_2_FPEF score.

## 1. Introduction

Heart failure with preserved ejection fraction (HFpEF) has become more prevalent in recent years, accounting for more than half of all heart failure cases. This rise is largely driven by population aging and the growing burden of comorbidities, such as diabetes, hypertension (HTN), and obesity [1]. These patients develop typical symptoms of heart failure, despite a preserved or near-normal ejection fraction, reflecting complex structural, functional, and systemic alterations that complicate timely diagnosis [2].

To help in HFpEF identification, several diagnostic scoring systems have been proposed, of which the HFA-PEFF algorithm and the H_2_FPEF are the most commonly used [3]. The HFA-PEFF algorithm integrates functional criteria: elevated left ventricular (LV) filling pressures, the presence of pulmonary hypertension, or reduced global longitudinal strain (GLS); morphological features: left atrial enlargement or left ventricular hypertrophy; and biomarker evidence in the form of elevated natriuretic peptides [4]. In contrast, the H_2_FPEF score relies on clinical and echocardiographic parameters, including age, hypertension and use of antihypertensive medication, body mass index (BMI), history of atrial fibrillation, and elevated LV filling pressures or pulmonary hypertension, without incorporating biomarkers [5].

Among these approaches, the HFA-PEFF algorithm uniquely assesses LV longitudinal function via GLS. However, GLS measurement requires high-quality echocardiographic imaging and specialized software, limiting its routine applicability. Simpler echocardiographic indices such as mitral annular plane systolic excursion (MAPSE) and myocardial systolic velocity (S’), obtained via pulsed-wave tissue Doppler imaging, offer accessible and time-efficient alternatives for evaluating LV longitudinal function [6,7].

Autonomic nervous system dysfunction represents a key, yet under-recognised component of heart failure pathophysiology, particularly in HFpEF. Sympathetic overactivation and impaired parasympathetic tone contribute to exercise intolerance, inefficient oxygen utilisation, and adverse outcomes [8,9].

Post-exercise HRR has emerged as a clinically relevant marker of autonomic function, with impaired HRR associated with increased mortality and reduced cardiopulmonary reserve [10].

Recently, greater attention has been directed toward the Heart Rate Recovery Index (HRRI), a novel parameter derived from exercise testing that integrates both heart rate acceleration time (AT) and deceleration time (DT) [11]. By incorporating both chronotropic response and recovery metrics, HRRI provides a more comprehensive assessment of autonomic regulation than conventional single-time-point HRR measures.

Therefore, the present study aims to investigate the role of HRRI as a functional marker in patients with preserved left ventricular ejection fraction, with a specific focus on HFpEF. Unlike our previous analyses that evaluated HRRI across the full spectrum of heart failure phenotypes [12], this study focuses exclusively on HFpEF and explores its association with early structural and functional alterations, including left atrial remodelling and longitudinal systolic dysfunction.

## 2. Materials and Methods

### 2.1. Study Design and Population

This prospective, dual-center observational cohort study was conducted at the Institute of Cardiovascular Diseases Timisoara and the County Clinical Emergency Hospital of Sibiu between October 2023 and June 2025. It included consecutive patients referred for clinically indicated exercise testing, who were subsequently categorized into HFpEF and non-heart failure groups for comparative analysis.

The general study framework, including exercise testing procedures, was similar to that previously reported by our group in an earlier HRRI-focused investigation [12].

Inclusion criteria: Eligible participants were adults aged 18 years or older who were referred for clinically indicated cardiac exercise testing.

Individuals with a previously established diagnosis of HFpEF and individuals without heart failure were referred for exercise testing due to other cardiovascular conditions such as hypertension, chronic coronary syndromes, or rhythm disturbances.

The following conditions were exclusion criteria: acute cardiovascular conditions (including acute coronary syndrome, myocarditis, endocarditis, or pulmonary embolism), severe systemic illness (such as sepsis, respiratory failure, end-stage renal disease, or advanced hepatic dysfunction), musculoskeletal or neurological disorders limiting exercise performance, and the occurrence of significant arrhythmias during testing that made HRRI determination unreliable. Patients underwent a thorough evaluation, including clinical examination, echocardiography, and standardized cyclo-ergometer exercise testing.

The study was conducted in accordance with the principles of the Declaration of Helsinki and was approved by the local Ethics Committees (approval no. 62/30.10.2023, revision 2024-Timisoara and 5046/29.02.2024-Sibiu). Written informed consent was obtained from all participants before enrolment.

### 2.2. Baseline Demographic Data

This study involved patients who previously had a diagnosis of heart failure (HF) as well as those without HF who had a preserved left ventricular ejection fraction (LVEF ≥ 50%). Individuals without HF were enrolled due to several indications for exercise testing: suspicion of coronary artery disease, history of syncope, hypertension, and arrhythmias.

A diagnosis of HFpEF was made in accordance with current European Society of Cardiology (ESC) guidelines, and symptoms were classified according to the New York Heart Association (NYHA) [13].

Natriuretic peptide measurements were obtained selectively, specifically in patients without a prior HF diagnosis who presented with signs or symptoms suggestive of HF at the time of exercise testing referral. Patients with an established HFpEF diagnosis had prior natriuretic peptide data available as part of their diagnostic workup.

The H_2_FPEF score was calculated after enrollment for research purposes only and was not used to establish the diagnosis.

In the current HFpEF-focused cohort, recorded comorbidities included arterial hypertension, chronic coronary syndromes, atrial fibrillation or flutter, and the presence of an implantable cardiac electronic device (CIED). Participants with CIEDs underwent device interrogation before testing to ensure sinus rhythm and a valid heart rate measurement. Device type and programming were recorded for documentation purposes but were not included in statistical analyses.

In addition, comorbidities such as obesity, type 2 diabetes mellitus, chronic kidney disease, obstructive sleep apnea, and asthma were also recorded.

Medication use within 24 h was noted, focusing on chronotropic drugs such as beta-blockers and antiarrhythmics due to their effect on heart rate response and recovery.

Laboratory testing was performed to evaluate comorbid conditions and identify contraindications to exercise, but the results were not included in the statistical analysis.

### 2.3. Exercise Testing and HRRI Calculation

Exercise testing was conducted using a semi-recumbent cycle ergometer (Ergoselect 1200 or GE e-Bike EL Ergoline GmbH, Bitz, Germany) following a modified Bruce protocol. The test began at a baseline workload of 25 W, increasing by 25 W at each 2 min stage, with adjustments based on individual patient tolerance. Baseline measurements of heart rate, blood pressure, and a standard 12-lead ECG were recorded before testing, and only participants in sinus rhythm were included.

During exercise, blood pressure was automatically monitored at each stage using the ergometer’s integrated sphygmomanometer, while continuous 12-lead ECG ensured ongoing assessment of heart rate and rhythm. The recovery phase consisted of one minute of active cycling at 0 W, followed by a passive seated cooldown.

**Figure 1:**
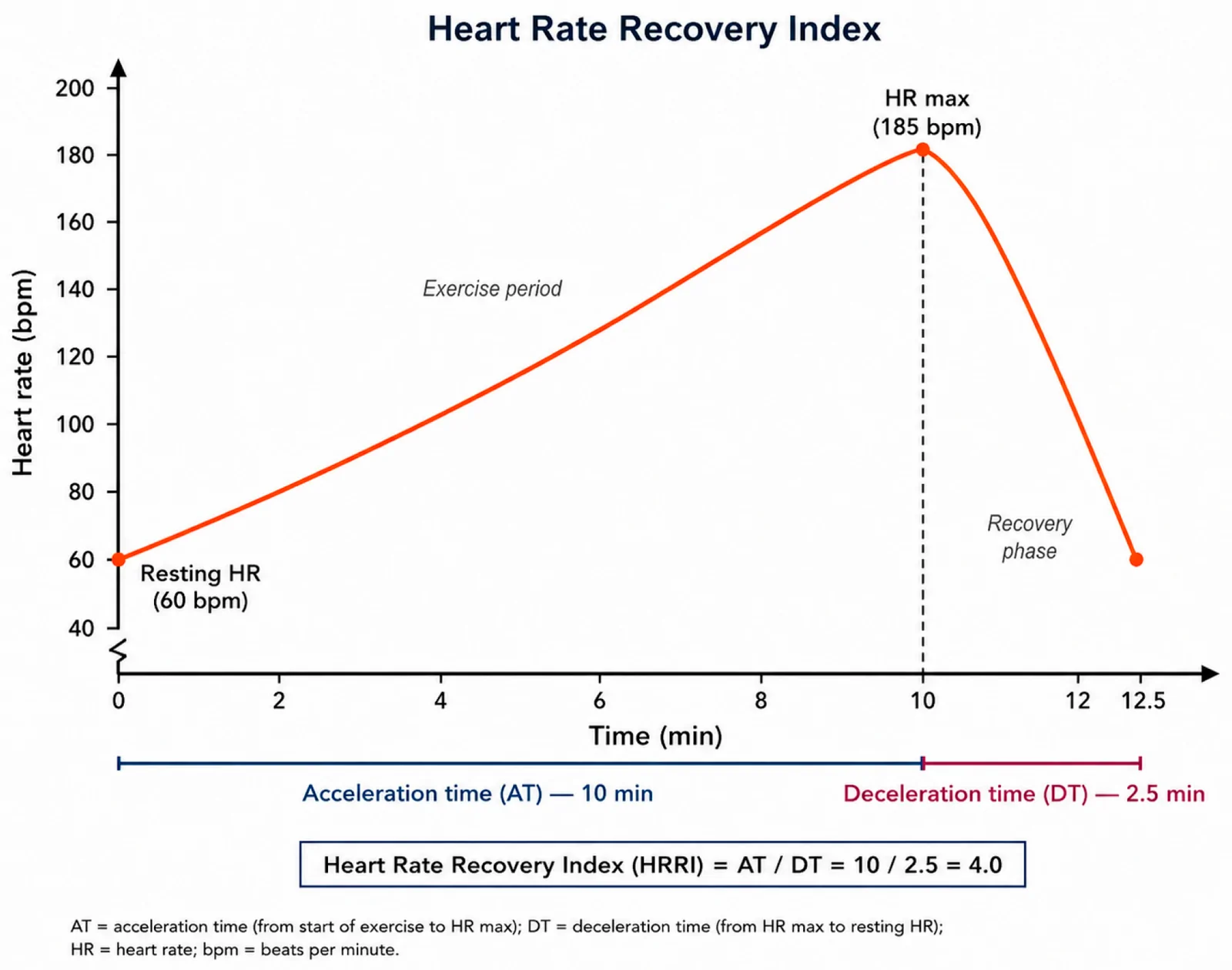
The HRRI Curve.

The following variables were evaluated during exercise testing, following the protocol previously described in our study on heart rate recovery in heart failure [12]:

– HRRI: Calculated manually as the ratio of heart rate acceleration time (AT) to deceleration time (DT). AT was defined as the interval from exercise onset to peak heart rate (HRmax), and DT as the time from HRmax to return to baseline heart rate (Figure 1).
– Peak Heart Rate and Age-Predicted Maximum Heart Rate (APMHR): Recorded to assess chronotropic response.
– Heart Rate Reserve (HRR): Calculated as the difference between HRmax and resting heart rate.
– Peak Blood Pressure: Maximal systolic and diastolic values during exercise.
– Exercise Capacity: Expressed in metabolic equivalents (METs).

### 2.4. Echocardiographic Assessment

Transthoracic echocardiography was performed prior to exercise testing using a Vivid 9 (GE Healthcare) or a Philips EPIQ 7 system, following standard imaging planes and techniques in accordance with current echocardiographic guidelines.

Left ventricular size was assessed by end-diastolic diameter, and hypertrophy was determined based on wall thickness. Global systolic function was quantified using the modified Simpson’s biplane method. Longitudinal systolic function was measured using mitral annular plane systolic excursion (MAPSE) and tissue Doppler S′ velocity at the septal and lateral mitral annulus.

Left ventricular diastolic dysfunction was graded according to the ASE/EACVI recommendations. Grade I (impaired relaxation) was defined by a mitral inflow E/A ratio ≤ 0.8 with a peak E-wave velocity ≤ 50 cm/s. Patients with E/A ≤ 0.8 and E > 50 cm/s or E/A > 0.8 to <2 underwent further assessment using average E/e′ ratio (>14), left atrial volume index (>34 mL/m^2^), and peak tricuspid regurgitation velocity (>2.8 m/s); Grade II was diagnosed when at least two of these three parameters were positive. Grade III (restrictive filling) was defined by an E/A ratio ≥ 2 [14]. For the ROC analysis, diastolic dysfunction was subsequently considered as a dichotomous variable (present/absent), irrespective of its severity grade.

Left atrial size was assessed at end-systole, and LAVI was calculated by indexing left atrial volume to body surface area.

Valvular heart disease was evaluated according to European Association of Cardiovascular Imaging recommendations, and sPAP was estimated from tricuspid regurgitation velocity combined with inferior vena cava assessment [15].

These echocardiographic methods were applied as previously described in our study on heart rate recovery in heart failure, with adaptations for the HFpEF cohort [12].

### 2.5. H_2_FPEF Score Calculation

The H_2_FPEF score, which combines clinical and echocardiographic parameters, was used to estimate the likelihood of HFpEF in each patient and was originally proposed by Reddy et al. [5]. The score includes the following components:

– Heavy (BMI > 30 kg/m^2^): 2 points
– Hypertensive (use of ≥2 antihypertensive medications): 1 point
– Atrial Fibrillation (paroxysmal or persistent): 3 points
– Pulmonary Hypertension (estimated PASP > 35 mmHg): 1 point
– Elder (age > 60 years): 1 point
– Filling Pressure (E/e′ > 9): 1 point

The H_2_FPEF score ranges from 0 to 9 points, with interpretation based on the original classification: 0–1 points indicate a low probability of HFpEF, 2–5 points suggest an intermediate probability, and 6–9 points correspond to a high likelihood of HFpEF.

### 2.6. Statistical Analysis

#### 2.6.1. Bivariate Analysis

Categorical variables were summarized with frequencies and percentages, while continuous variables were described using means, standard deviations, minimum and maximum values, interquartile ranges, and 95% confidence intervals. The distribution of continuous variables was evaluated for normality using the Kolmogorov–Smirnov or Shapiro–Wilk tests. Group comparisons for categorical variables were conducted with the Chi-square test or Fisher’s exact test, as appropriate. Continuous variables were compared with the independent samples t-test when normally distributed, or the Mann–Whitney U-test when normality was not present. For comparisons involving more than two groups, one-way analysis of variance (ANOVA) was used for normally distributed data, and the Kruskal–Wallis test was applied when the normality assumption was violated.

A significance threshold of α = 0.05 was applied for all bivariate analyses and for determining which variables were eligible for inclusion in logistic regression models. Data management, visualization, and analyses were conducted using Microsoft Excel^®^ and SPSS^®^ version 21.0.0.0.

#### 2.6.2. Logistic Regression

Multiple logistic regression models were built using variables that showed significant links to heart failure, regardless of ejection fraction class, in the bivariate analyses. Variable selection used an iterative stepwise approach, guided by clinical parameters and findings from echocardiographic and exercise testing. Continuous variables were mean-centered to minimize multicollinearity, and categorical variables were transformed into dummy variables. To improve the robustness of the results, bootstrapping with 1000 resamples was conducted, and 95% confidence intervals for the regression coefficients were calculated using the bias-corrected and accelerated (BCa) method. Only variables that significantly contributed to predicting heart failure (*p* < 0.05, with the 95% confidence interval of the coefficient excluding zero) were included in the final model.

## 3. Results

### 3.1. Baseline Characheristics and Bivariate Analysis

A total of 241 patients met the inclusion criteria and were enrolled in the study, including 138 patients with heart failure with preserved ejection fraction (HFpEF) and 103 patients without heart failure (non-HF). The age of participants ranged from 22 to 82 years, with a mean age of 62.47 ± 10.24 years in the HFpEF group versus 50.92 ± 11.69 years in the non-HF group (*p* < 0.01). Patients with HFpEF were slightly more overweight (*p* < 0.01) and had a higher prevalence of chronic coronary syndrome (CCS, *p* = 0.022), hypertension (HTN, *p* < 0.01), atrial fibrillation or atrial flutter (AF/AFL, *p* < 0.01), cardiac implantable electronic device (CIED) implantation (*p* < 0.01), and chronic kidney disease (CKD, *p* < 0.01) compared to the non-HF group.

Regarding baseline therapy, HFpEF patients were more frequently treated with angiotensin-converting enzyme inhibitors (ACEi, *p* < 0.01), angiotensin receptor blockers (ARB, *p* < 0.01), dihydropyridine calcium channel blockers (DHP-CCB, *p* < 0.01), mineralocorticoid receptor antagonists (MRA,*p* < 0.01), angiotensin receptor–neprilysin inhibitors (ARNI, *p* < 0.01), diuretics (*p* < 0.01), beta-blockers (*p* < 0.01), amiodarone (*p* = 0.020), sodium–glucose cotransporter-2 inhibitors (SGLT2i, *p* < 0.01), and statins (*p* < 0.01).

Of note, 12 patients had a prior diagnosis of HFrEF with subsequent recovery of ejection fraction and were classified as HFpEF at the time of enrolment, based on a current LVEF ≥ 50% and ESC criteria.

The H_2_FPEF score was significantly higher in patients with HFpEF compared with those without HF (*p* < 0.01).

Baseline characteristics of the two groups are summarized in Table 1.

Baseline echocardiographic parameters are summarized in Table 2. Left ventricular end-diastolic volume was higher in the HFpEF group (*p* < 0.01). Markers of left atrial remodelling were significantly increased in HFpEF patients, including left atrial area and indexed left atrial volume (all *p* < 0.01).

Parameters reflecting diastolic function were significantly impaired in the HFpEF group. Septal and lateral e′ velocities were significantly lower, while E/e′ ratios were significantly higher compared with the non-HF group (all *p* < 0.01), indicating elevated left ventricular filling pressures.

Furthermore, indices of longitudinal systolic function were reduced in the HFpEF group, including septal and lateral S′ velocities as well as septal and lateral MAPSE (all *p* < 0.01). Systolic pulmonary artery pressure was also higher in HFpEF patients (*p* < 0.01).

A comparison of exercise parameters is shown in Table 3. During exercise testing, HFpEF patients had a lower HRRI index (*p* < 0.01), lower acceleration time (AT, *p* < 0.01), lower heart rate reserve (HR reserve, *p* < 0.01), and lower exercise capacity (*p* < 0.01) compared to non-HF patients.

Patients with an H_2_FPEF score > 4 had significantly lower HRRI values than those with an H_2_FPEF score ≤ 4 (median [IQR]: 1.89 [0.52], *n* = 54 vs. 2.22 [1.00], *n* = 187; Mann–Whitney U test, *p* < 0.01).

Table 4 shows correlations between HRRI and clinical, echocardiographic and exercise test parameters.

Table 5 displays the correlations between HRRI and the diastolic dysfunction grade.

**Figure 2:**
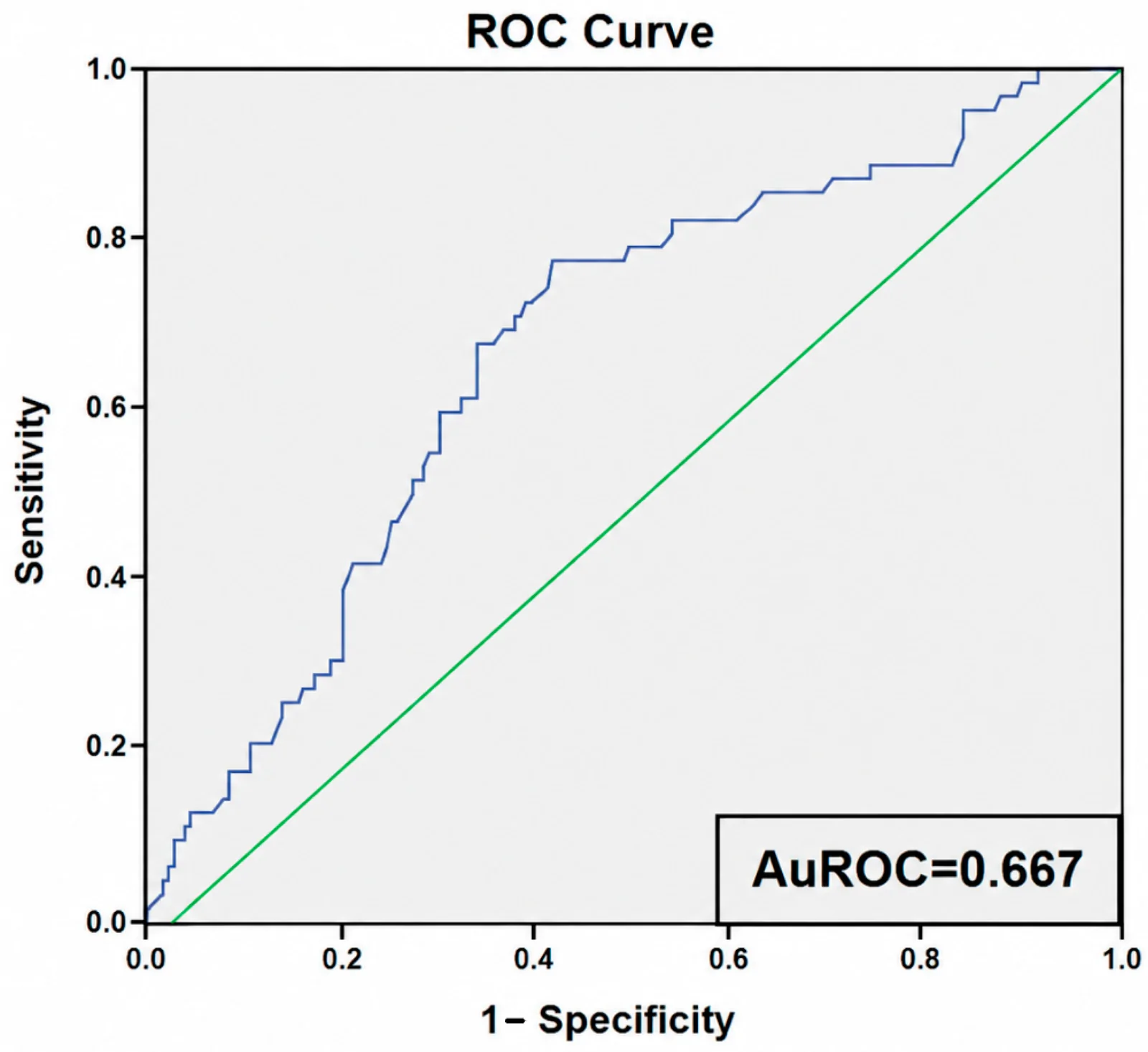
ROC Curve for HRRI Predicting Diastolic Dysfunction.The blue line represents the ROC curve, while the green diagonal line represents the reference line (AuROC = 0.5).

Figure 2 illustrates the ROC curve for HRRI predicting diastolic dysfunction. Lower HRRI values were associated with the presence of diastolic dysfunction. HRRI demonstrated modest discriminative ability, with an AUC of 0.667 (95% CI: 0.591–0.744; *p* < 0.001). We subsequently assessed the relationship between baseline medical therapy to identify a potentially medication-related influence on the chronotropic response and HRRI values. For each medication class, HRRI values were compared between patients receiving and not receiving the respective therapy. The results are summarized in Table 6.

### 3.2. Logistic Regression and Diagnostic Performance of HRRI for HFpEF Identification

**Figure 3:**
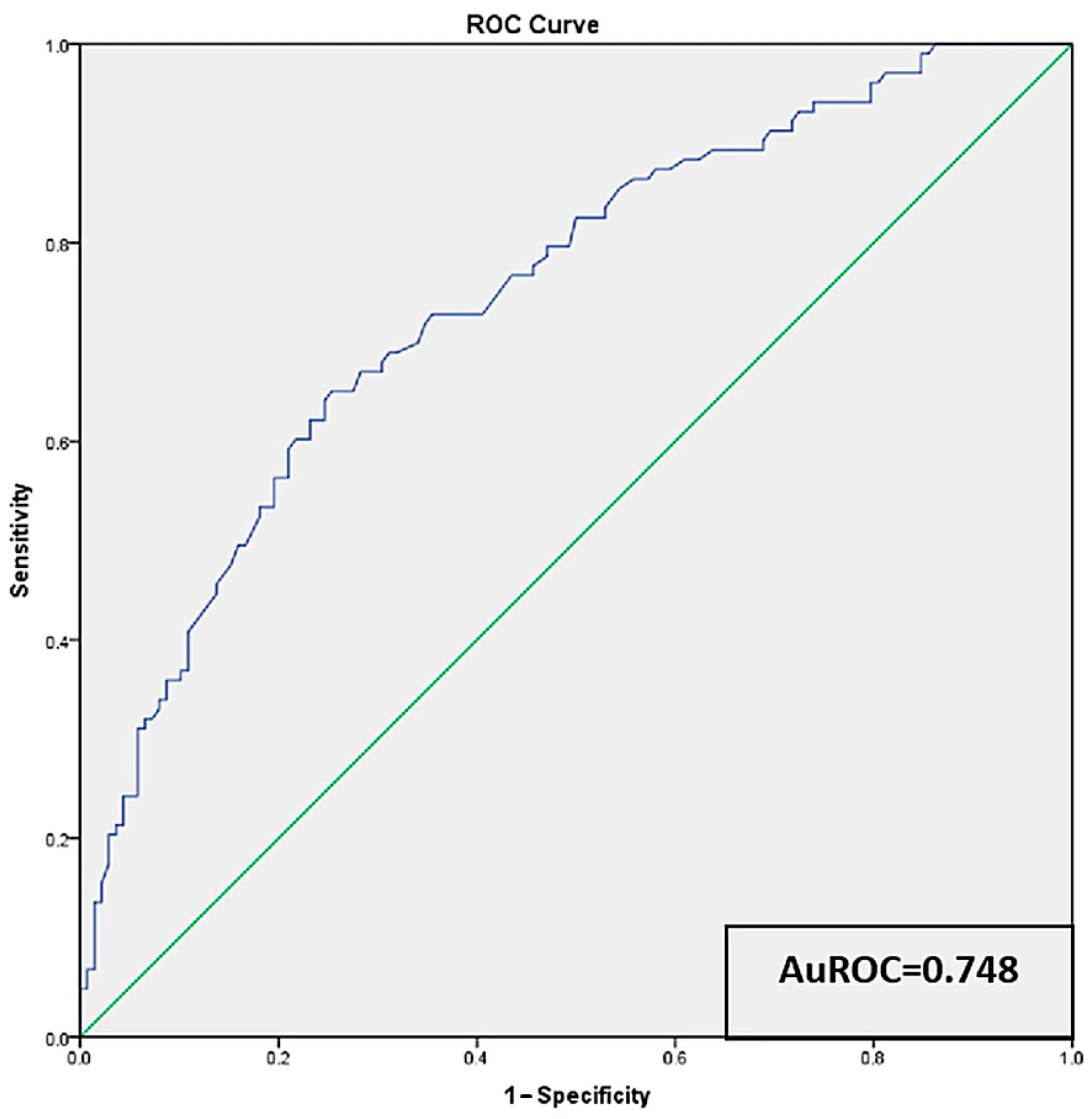
ROC Curve for HRRI Predicting HFpEF.The blue line represents the ROC curve, while the green diagonal line represents the reference line (AuROC = 0.5).

The ROC analysis evaluating the ability of HRRI to diagnose HFpEF yielded an AUC of 0.748 with a standard error of 0.032, as shown in Figure 3.

The 95% confidence interval for the AUC ranged from 0.686 to 0.810. The asymptotic significance was *p* < 0.01, indicating that the discriminative capacity of HRRI was statistically significant. The optimal HRRI cut-off point for diagnosing HFpEF in this dataset was 2.25, yielding a Youden Index of 0.397, with a sensitivity of 65% and specificity of 74.7%.

**Figure 4:**
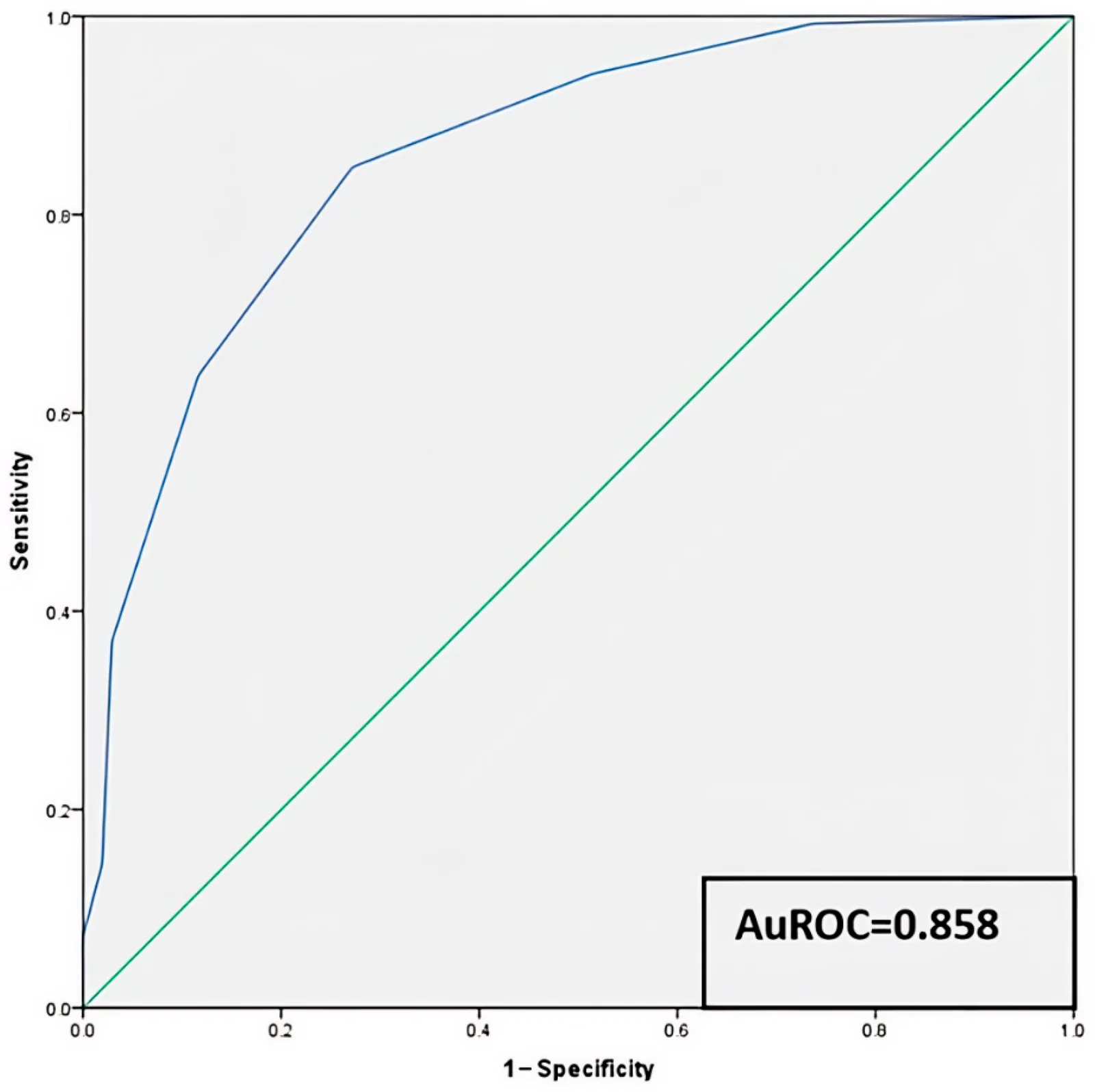
ROC Curve for the H_2_FPEF Score Predicting HFpEF.The blue line represents the ROC curve, while the green diagonal line represents the reference line (AuROC = 0.5).

Figure 4 displays the ROC Curve for the H_2_FPEF Score in predicting HFpEF. The AUC is 0.858, and the 95% confidence interval ranges from 0.811 to 0.906. The result is statistically significant, with an asymptotic *p*-value < 0.01.

We developed a binary logistic regression model to predict the presence of HFpEF based on the H_2_FPEF score and mean-centered HRRI. The model demonstrated an overall classification accuracy of 82.2%, with a sensitivity of 85.5% for identifying HFpEF and a specificity of 77.7% for correctly identifying patients without HF. Calibration was adequate, as evidenced by a non-significant Hosmer–Lemeshow test (*p* = 0.137).

Table 7 summarizes the regression coefficients, *p*-values, and bias-corrected and accelerated (BCa) 95% confidence intervals based on 1000 bootstrap samples.

Based on the probabilities predicted by the logistic regression model, an ROC analysis was performed. In this context, the AUROC reflects the model’s ability to discriminate between cases with and without HFpEF, using the predicted probabilities derived from the included predictors (HRRI and H_2_FPEF).

**Figure 5:**
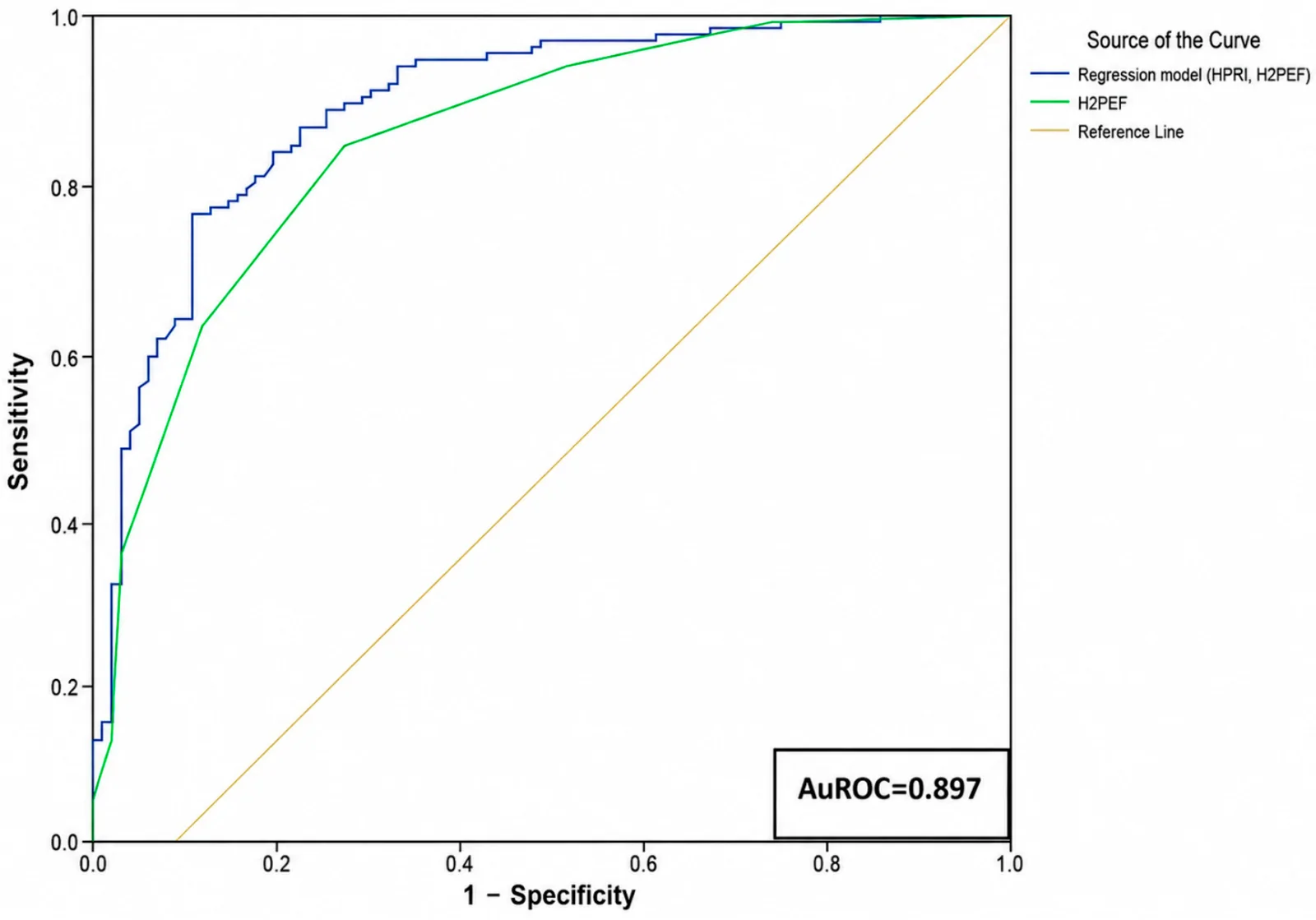
ROC curves comparing the combined HRRI–H_2_FPEF model with the H_2_FPEF score alone for HFpEF prediction.

The AUROC for the logistic regression model was 0.897, with a 95% confidence interval ranging from 0.856 to 0.937 and a statistically significant *p*-value (*p* < 0.01). Figure 5 illustrates the ROC curves of the logistic regression model incorporating both HRRI and H_2_FPEF, compared to the H_2_FPEF score alone.

Table 8 summarizes the regression coefficients, *p*-values, and bias-corrected and accelerated (BCa) 95% confidence intervals based on 1000 bootstrap samples. None of the evaluated medication classes retained statistical significance after multivariable adjustment, whereas HRRI and the H_2_FPEF score remained significantly associated with HFpEF.

To address the potential confounding effect of medical therapy, we conducted an additional binary logistic regression analysis with HFpEF diagnosis as the dependent variable. The initial model included HRRI and the H2FPEF score as predictor variables. Furthermore, medication classes that showed significant associations with HRRI in the preceding bivariate analysis were added to the model to assess whether HRRI remained independently associated with HFpEF after adjustment for treatment-related factors.

## 4. Discussion

The present study provides evidence that HRRI may represent a useful functional marker in patients with HFpEF. HFpEF patients exhibited significantly lower HRRI values than individuals without heart failure, and lower HRRI was associated with a higher H_2_FPEF score, greater left atrial volume, higher E/e′ ratio, impaired longitudinal left ventricular systolic function, and reduced exercise capacity.

Importantly, HRRI demonstrated good discriminative ability for HFpEF, with an AUC of 0.748 (95% CI 0.686–0.810, *p* < 0.001), while the combination of HRRI with the H_2_FPEF score further improved classification.

In our current study, we evaluated HFpEF patients in comparison to individuals without HF. Our study cohort comprised 241 patients, of whom 138 had HFpEF. Consistent with recognized HFpEF phenotypes [16], our HFpEF patients were older, hypertensive, and more likely to have chronic kidney disease, atrial arrhythmias, and coronary artery disease.

Clinical evaluation incorporated the H_2_FPEF score alongside structural assessment of left atrial remodelling and LV longitudinal systolic function, complemented by HRRI as a functional parameter reflecting autonomic recovery and chronotropic competence.

Patients with HFpEF had significantly lower HRRI compared with individuals without heart failure. These findings are consistent with previous reports, with reduced HRRI reflecting autonomic dysfunction in heart failure and being associated with elevated filling pressures. Lower peak workload and METS further highlight how impaired HRRI relates to diminished functional capacity and exercise tolerance in this population [12,17]. In our previous study, HRRI was significantly reduced in patients with heart failure and discriminated among HF phenotypes, including HFpEF, HFmrEF, and HFrEF [12]. Building on these results, the present study further explores the potential role of HRRI in HFpEF, a condition in which early detection remains particularly challenging [18].

In our ROC analysis, HRRI demonstrated good discriminative ability for identifying HFpEF, with an AUC of 0.748 (95% CI 0.686–0.810, *p* < 0.001), indicating that HRRI differentiates HFpEF patients from individuals without HF in our cohort (Figure 3). The optimal HRRI cut-off of 2.25 yielded a sensitivity of 65% and specificity of 74.7%, supporting its potential utility as a screening tool in clinical practice. Notably, these results are consistent with our recently published data, where an optimal HRRI cut-off of 2.225 was identified to distinguish HF patients from non-HF individuals (AUC ≈ 0.753), highlighting similar discriminatory performance [12].

The H_2_FPEF score demonstrated even greater discriminative ability, achieving an AUC of 0.858 (95% CI 0.811–0.906, *p* < 0.01), as indicated in Figure 4. Although the H_2_FPEF score integrates established clinical and echocardiographic determinants of HFpEF, it does not capture autonomic regulation during exercise and recovery. Incorporating HRRI into a binary logistic regression model alongside the H_2_FPEF score additionally improved classification (Figure 5). Table 7 shows that the H_2_FPEF score was positively associated with HFpEF (β = 0.978, *p* < 0.01), whereas HRRI was negatively associated (β = −1.097, *p* < 0.01), demonstrating that reduced HRRI contributes to HFpEF risk prediction.

HRRI reflects the interplay between sympathetic withdrawal and parasympathetic reactivation post-exercise, representing a physiological dimension distinct from, yet complementary to, structural and hemodynamic abnormalities. The improved discrimination of the combined HRRI–H_2_FPEF model supports the contribution of autonomic dysfunction beyond conventional structural and hemodynamic markers.

The H_2_FPEF score was significantly higher in HFpEF patients compared to non-HF individuals (median 4 vs. 2, IQR 2, *p* < 0.01). Moreover, patients with an H_2_FPEF score of >4 exhibited significantly lower HRRI values, suggesting impaired autonomic regulation in those with a higher likelihood of HFpEF.

A previously published study revealed that patients with an H_2_FPEF score > 4 are associated with impaired left atrial function in the acute setting of HFpEF. Additionally, the score served as an indicator of diastolic impairment, as well as a prognostic factor [19].

Recent evidence indicates that higher H_2_FPEF scores are associated with reduced exercise capacity in HFpEF patients, reflecting the score’s ability to capture functional limitations in addition to diagnostic risk [3]. In addition, another study has shown that primary care patients with an H_2_FPEF score ≥ 4 had a higher prevalence of HFpEF and experienced worse outcomes, highlighting the utility of this cut-off for risk stratification in this population [20].

Our current study revealed that HFpEF patients had significantly larger left atrial volumes. Recent literature has outlined the concept of atrial cardiomyopathy, in which atrial abnormalities may occur independently of LV dysfunction or, more commonly, overlap with and exacerbate HFpEF. In such cases, determining which process developed first remains thought-provoking [21,22].

Moreover, HFpEF patients tend to have higher average E/e’ values, indicating increased ventricular filling pressures [23]. These elevated pressures are a key feature of HFpEF and are a central part of the H_2_FPEF score.

Table 4 reveals correlations between HRRI and important clinical, echocardiographic and exercise test parameters, reflecting both exercise capacity and overall cardiovascular status. Interestingly, HRRI was negatively correlated with E/e’ ratio, LAVi, and sPAP, suggesting that impaired autonomic function is related to high LV filling pressures and atrial remodelling. These findings are consistent with previously published data that exhibit a correlation with impaired dysautonomia, as measured by HRR and elevated filling pressures [24].

A recent study has demonstrated that autonomic dysfunction, determined by reduced HRR at 120 s post-exercise, is independently associated with impaired left atrial conduit function, suggesting a link between autonomic imbalance and atrial remodelling [25].

HFpEF patients exhibited decreased longitudinal ventricular contraction, as measured by MAPSE and TDI-derived mitral annulus systolic velocities (S wave), reflecting early and subtle impairments in longitudinal LV contraction. This finding is clinically relevant, as previous studies have demonstrated the prognostic utility of the S wave and MAPSE across diverse settings, from predicting mortality in sepsis to identifying patients at risk of malignant arrhythmias with CIEDs, even independently of LVEF [26,27,28].

The correlation between HRRI and MAPSE suggests a modest association between HRR dynamics and longitudinal systolic function. MAPSE is a simple and robust marker of longitudinal LV performance, correlating well with LVEF and allowing early detection of subtle systolic dysfunction [28,29].

Conversely, effort capacity, as measured by METS and HR reserve, is positively associated with HRRI. An earlier study has shown that heart rate recovery has proven to be independently associated with peak oxygen consumption (VO_2_) in HF patients with atrial fibrillation undergoing exercise testing [30].

Chronotropic agents were not withheld before exercise testing. This approach aimed to assess HRRI under routine clinical conditions and to reflect patients’ habitual physiological status during standard medical therapy.

Several cardiovascular medications may influence chronotropic response and post-exercise heart rate kinetics. HRRI values were compared between patients receiving and not receiving each medication class. Significant associations were observed for ACE inhibitors, ARBs, dihydropyridine calcium channel blockers, SGLT2 inhibitors, diuretics, beta-blockers, and amiodarone.

To assess whether these associations reflected confounding rather than independent effects, we performed an additional logistic regression with HFpEF diagnosis as the dependent variable, incorporating both HRRI and H_2_FPEF score as predictors, followed by sequential addition of the implicated medication classes. None retained statistical significance after multivariable adjustment, while HRRI and H_2_FPEF score remained significant, consistent with confounding by indication.

In our previous study, beta-blockers were not significantly associated with HRRI, whereas ivabradine demonstrated a significant association with reduced HRRI values. This discrepancy likely reflects the more heterogeneous composition of the previous cohort, which included patients across the full spectrum of heart failure phenotypes (HFpEF, HFmrEF, HFrEF) in addition to individuals without heart failure. By contrast, the present analysis focused specifically on HFpEF, a population characterized by a distinct clinical and comorbidity profile, which may have altered the relationship between baseline medical therapy and HRRI values. Nevertheless, HRRI remained significantly associated with HFpEF even after adjustment for baseline medical therapy, supporting its potential utility as a functional marker in this population.

## 5. Study Limitations

This study has several limitations. First, this was a dual-center observational study with a relatively modest sample size, which may limit the generalizability of the findings. Second, biomarkers commonly used in HFpEF assessment, such as NT-proBNP, were not incorporated into the statistical analysis. Moreover, invasive exercise hemodynamic assessment, exercise stress echocardiography, and systematic use of the HFA-PEFF algorithm were not routinely performed due to their time-consuming nature, limited availability, and need for specialized expertise. Future studies incorporating these methods may better validate the role of HRRI in patients with HFpEF. Finally, external validation of the proposed model was not available.

## 6. Conclusions

In patients with preserved left ventricular ejection fraction, HRRI was significantly reduced in those with HFpEF and was consistently associated with exercise performance, H_2_FPEF score, and echocardiographic markers of diastolic and longitudinal systolic dysfunction, including LAVI and MAPSE. The addition of HRRI to the H_2_FPEF score improved discrimination for HFpEF identification, suggesting that HRRI may provide complementary functional information beyond conventional structural and clinical parameters. Given its simplicity and integration into routine exercise testing, HRRI may serve as a practical complementary marker for HFpEF identification and functional assessment. Further studies with external validation are needed to confirm these findings and establish their clinical utility.

## Figures and Tables

**Table 1:** Baseline characteristics, underlying disease, treatment and echocardiographic parameters.

Variable			HFpEF (138)	No HF (*n* = 103)	*p*-Value
Age (years) Mean ± SD			62.47 ± 10.24	50.92 ± 11.69	<0.01
Male, *n* (%)			80 (58%)	53 (51.5%)	0.314
BMI (kg/m^2^) Mean ± SD			29.9154 ± 5.30	28.14 ± 4.92	<0.01
Smokers, *n* (%)			30 (21.7%)	22 (21.4%)	1.000
CCS, *n* (%)			63 (45.7%)	32 (31.1%)	0.022
HTN, *n* (%)			126 (91.3%)	60 (58.3%)	<0.01
History of AF/AFL, *n* (%)			35 (25.4%)	10 (9.7%)	<0.01
CIED (*n*%)			18 (13%)	3 (2.9%)	<0.01
NYHA Class	I		27 (19.5%)	-	
	II		109 (78.9%)		
	III		2 (1.4%)		
Respiratory disease, *n* (%)		Asthma	3 (2.2%)	4 (3.9%)	0.434
		OSA	16 (11.6%)	8 (7.8%)	0.326
Chronic Kidney Disease, *n* (%)			15 (10.9%)	1 (1.0%)	<0.01
H_2_FPEF Score (Median + IQR)			4 (2)	2 (3)	<0.01
Baseline Therapy, *n* (%)					
ACEi			63 (45.7%)	29 (28.2%)	<0.01
ARB			41 (29.7%)	16 (15.5%)	<0.01
Non-DHP CCB			3 (2.2%)	3 (2.9%)	0.716
DHP-CCB			50 (36.2%)	16 (15.5%)	<0.01
MRA			33 (23.9%)	7 (6.8%)	<0.01
ARNI			12 (8.7%)	0 (0.0%)	<0.01
Diuretics			68 (49.3%)	19 (18.4%)	<0.01
Betablockers			95 (68.8%)	51 (49.5%)	<0.01
Ivabradine			6 (4.3%)	3 (2.9%)	0.561
Amiodarone			15 (10.9%)	3 (2.9%)	0.020
SGLT2i			31 (22.5%)	4 (3.9%)	<0.01
Statin			120 (87.0%)	58 (56.3%)	<0.01

ACEi—angiotensin-converting enzyme inhibitors; AF—atrial fibrillation; AFL—atrial flutter; ARB—angiotensin receptor blockers; ARNI—angiotensin receptor–neprilysin inhibitor; BMI—body mass index; CCB—calcium channel blockers; CCS—chronic coronary syndrome; CIED—cardiac implantable electronic device; DHP-CCB—dihydropyridine calcium channel blockers; IQR—interquartile range; HFpEF—heart failure with preserved ejection fraction; HTN—hypertension; MRA—mineralocorticoid receptor antagonists; Non-DHP CCB—non-dihydropyridine calcium channel blockers; OSA—obstructive sleep apnea; SGLT2i—sodium–glucose cotransporter-2 inhibitors.

**Table 2:** Main Echocardiographic parameters and comparison between groups.

Baseline Echocardiography Mean ± StdDev [Median ± IQR]/*n* (%)		HFpEF (138)	No HF (*n* = 103)	*p*-Value
LVEDV (mL)		100 (36.25)	90 (26)	<0.01
LA Area (cm^2^)		21.5 (7)	18.3 (5)	<0.01
LAVi (mL/m^2^)		36.68 ± 11.45 *	28.13 ± 9.37	<0.01
E (m/s)		0.69 (0.25)	0.72 (0.23)	0.40
A (m/s)		0.80 (0.22)	0.73 (0.23)	<0.01
E/A ratio		0.80 (0.35)	0.94 (0.52)	<0.01
e′ septal (m/s)		0.07 (0.03)	0.09 (0.04)	<0.01
e′ lateral (m/s)		0.08 (0.04)	0.11 (0.05)	<0.01
E/e′ septal		10 (4)	7.6 (2.43)	<0.01
E/e′ lateral		8.25 (4.33)	6.56 (2.78)	<0.01
E/e′ mean		9.17 (3.78)	7.18 (2.41)	<0.01
Diastolic dysfunction	Type 1	102 (73.9%)	51 (49.5%)	<0.01
	Type 2	22 (15.9%)	2 (1.9%)	<0.01
	Type 3	3 (2.2%)	0	<0.01
TDI Sseptal (cm/s)		8 (2.25)	9 (2)	<0.01
TDI S lateral (cm/s)		9 (3)	10 (3)	<0.01
MAPSE septal (mm)		14 (4)	15 (3)	<0.01
MAPSE lateral (mm) *		15.30 ± 2.77	16.63 ± 2.24	<0.01
Mitral regurgitation (at least moderate)		38 (27.5%)	9 (8.7%)	<0.01
Tricuspid regurgitation		27 (19.6%)	5 (4.9%)	<0.01
sPAP (mmHg) *		28.58 ± 7.30	24.87 ± 4.99	<0.01

HF—heart failure; HFpEF—heart failure with preserved ejection fraction; LAVi—left atrial volume index; LVEDV—left ventricular end-diastolic volume; TDI S—peak systolic velocity by tissue Doppler imaging (lateral or septal). StdDev—Standard Deviation sPAP—systolic pulmonary arterial pressure; *—normal data distribution.

**Table 3:** Exercise parameters comparison between groups.

Variable	Descriptive Parameter	HFpEF (*n* = 138)	No HF (*n* = 103)	*p*-Value
AT	Median	325.20	432	<0.01
	IQR	128.50	189.60	
DT	Median	180	165	<0.01
	IQR	96	64	
HRRI	Median	1.93	2.50	<0.01
	IQR	0.74	1.09	
HR MAX	Median	106	131	<0.01
	IQR	26	26	
HR Start	Median	69	75	<0.01
	IQR	13	18	
HRReserve *	Mean	39.87	54.75	<0.01
	StdDev	15.04	14.95	
BP max	Median	170.50	181	<0.01
	IQR	40	37	
Stress (W)	Median	100	125	<0.01
	IQR	31.25	50	
METS	Median	6.90	8.30	<0.01
	IQR	2.43	3.10	

AT—Acceleration Time, DT—Deceleration Time, HRRI—Heart Rate Recovery Index, HR MAX—Maximum Heart Rate, HR Start—Resting Heart Rate at the beginning of the exercise test, HRReserve—Heart Rate Reserve (HR max—HR start), BP max—Maximum Systolic Blood Pressure during exercise, Stress (W)—Maximal Workload achieved during exercise (Watts), METS—Metabolic Equivalent of Task; *—normal data distribution.

**Table 4:** Correlations Between HRRI, Echocardiographic and Exercise Test Parameters (Spearman’s Rho).

Variable	Spearman Correlation Coefficient	*p*-Value
DT	−0.572	<0.01
AT	0.437	<0.01
Stress (W)	0.403	<0.01
Age	−0.396	<0.01
H_2_FPEF score	−0.288	<0.01
METS	0.270	<0.01
E/e’ (mean)	−0.238	<0.01
BSA	0.228	<0.01
HRReserve	0.214	<0.01
MAPSE (lateral)	0.176	0.017
MAPSE (septal)	0.172	<0.01
LAVi	−0.172	<0.01
BMI	0.139	0.031
TDI S (lateral)	0.130	0.058
sPAP	−0.118	0.090
TDI S (septal)	0.096	0.136

AT—acceleration time; BMI—body mass index; BSA—body surface area; DT—deceleration time; HRReserve—heart rate reserve; H_2_FPEF—Heart Failure with Preserved Ejection Fraction score; LAVi—left atrial volume index; e′—early diastolic mitral annular velocity (mean, lateral, or septal); MAPSE—mitral annular plane systolic excursion (lateral or septal); METS—metabolic equivalents; sPAP—systolic pulmonary artery pressure; Stress (W)—maximal workload in watts; TDI S—peak systolic velocity by tissue Doppler imaging (lateral or septal).

**Table 5:** HRRI correlations with the diastolic dysfunction grade.

**Variable**	**Descriptive** **Parameter**	**Diastolic Dysfunction Grade**			***p*-Value**
		**None**	1	**≥2**	
	Median	2.49	2.06	1.8	
	IQR	0.9	0.97	0.69	

HRRI—Heart rate recovery index; IQR—Interquartile range.

**Table 6:** HRRI variations according to baseline therapy.

Baseline Therapy	HRRI—On Therapy (Median [IQR])	HRRI w/o Therapy (Median [IQR])	*p*-Value
ACEi	2.02 [0.80]	2.22 [1]	0.027
ARB	2.04 [1.01]	2.15 [0.99]	0.03
Non-DHP CCB	2.44 [1.70]	2.12 [0.97]	0.367
DHP-CCB	1.95 [0.67]	2.19 [0.97]	0.005
MRA	2.04 [0.83]	2.14 [1.01]	0.364
ARNI	1.95 [1.02]	2.14 [0.97]	0.311
SGLT2i	1.97 [0.73]	2.15 [1.01]	0.011
Diuretics	2.04 [0.96]	2.19 [0.95]	0.035
Betablockers	2.06 [0.93]	2.19 [1]	0.047
Ivabradine	2.13 [0.97]	1.93 [1.15]	0.103
AA1C	2.43 [0.73]	2.08 [0.98]	0.145
Amiodarone	1.65 [0.92]	2.14 [0.96]	0.032

AA1C—Class IC antiarrhythmic agents; ACEi—Angiotensin-converting enzyme inhibitors; ARB—Angiotensin receptor blockers; ARNI—Angiotensin receptor–neprilysin inhibitors; DHP-CCB—Dihydropyridine calcium channel blockers; HRRI—Heart rate recovery index; IQR—Interquartile range; MRA—Mineralocorticoid receptor antagonists; Non-DHP CCB—Non-dihydropyridine calcium channel blockers; SGLT2i—Sodium–glucose cotransporter 2 inhibitors.

**Table 7:** Binary logistic regression model for predicting HFpEF based on H_2_FPEF score and mean-centered HRRI.

Variable	β	*p*	BCa 95% CI for β	
			Lower	Higher
H_2_FPEF	0.978	<0.01	0.706	1.384
HRRI (mean-centered)	−1.097	<0.01	−1.717	−0.675
Constant	−2.401	<0.01	−3.198	−1.806

HFpEF—Heart failure with preserved ejection fraction; HRRI—Heart Rate Recovery Index; β, regression coefficient; BCa CI, bias-corrected and accelerated confidence interval; CI, confidence interval.

**Table 8:** Multivariable Bootstrap Regression Analysis for Independent Predictors of HFpEF.

Variable	β	*p*	BCa 95% CI for β	
			Lower	Upper
H_2_FPEF	0.876	0.001	0.518	1.667
HRRI (mean-centered)	−1.066	0.001	−1.636	−0.759
ACEi	0.028	0.956	−1.074	1.177
ARB	−0.114	0.871	−1.334	1.227
DHP-CCB	−0.198	0.653	−1.292	0.872
SGLT2i	−1.262	0.081	−3.224	−0.226
Diuretics	−0.864	0.066	−1.901	−0.035
Betablockers	0.07	0.852	−0.79	1.122
Amiodarone	−0.982	0.172	−3.58	0.407
Constant	0.702	0.619	−2.898	21.185

ACEi—Angiotensin-converting enzyme inhibitors; ARB—Angiotensin receptor blockers; BCa—Bias-corrected and accelerated; CI—Confidence interval; DHP-CCB—Dihydropyridine calcium channel blockers; H_2_FPEF—Heart Failure with Preserved Ejection Fraction score; HRRI—Heart rate recovery index; SGLT2i—Sodium–glucose cotransporter 2 inhibitors.

## Data Availability

Data are available upon request due to restrictions (privacy and ethical reasons).

## References

[1] Omote K., Verbrugge F.H., Borlaug B.A. (2022). Heart Failure with Preserved Ejection Fraction: Mechanisms and Treatment Strategies. Annu. Rev. Med..

[2] Albani S., Zilio F., Scicchitano P., Musella F., Ceriello L., Marini M., Gori M., Khoury G., D’ANdrea A., Campana M. (2024). Comprehensive diagnostic workup in patients with suspected heart failure and preserved ejection fraction. Hell. J. Cardiol..

[3] Amanai S., Harada T., Kagami K., Yoshida K., Kato T., Wada N., Obokata M. (2022). The H2FPEF and HFA-PEFF algorithms for predicting exercise intolerance and abnormal hemodynamics in heart failure with preserved ejection fraction. Sci. Rep..

[4] Pieske B., Tschöpe C., de Boer R.A., Fraser A.G., Anker S.D., Donal E., Edelmann F., Fu M., Guazzi M., Lam C.S.P. (2019). How to diagnose heart failure with preserved ejection fraction: The HFA–PEFF diagnostic algorithm: A consensus recommendation from the Heart Failure Association (HFA) of the European Society of Cardiology (ESC). Eur. Heart J..

[5] Reddy Y.N.V., Carter R.E., Obokata M., Redfield M.M., Borlaug B.A. (2018). A Simple, Evidence-Based Approach to Help Guide Diagnosis of Heart Failure with Preserved Ejection Fraction. Circulation.

[6] Ozer P.K., Govdeli E.A., Demirtakan Z.G., Nalbant A., Baykiz D., Orta H., Bayraktar B.B., Baskan S., Umman B., Bugra Z. (2022). The relation of echo-derived lateral MAPSE to left heart functions and biochemical markers in patients with preserved ejection fraction: Short-term prognostic implications. J. Clin. Ultrasound.

[7] Shin H.-W., Kim H., Son J., Yoon H.-J., Park H.-S., Cho Y.-K., Han C.-D., Nam C.-W., Hur S.-H., Kim Y.-N. (2010). Tissue Doppler Imaging as a Prognostic Marker for Cardiovascular Events in Heart Failure with Preserved Ejection Fraction and Atrial Fibrillation. J. Am. Soc. Echocardiogr..

[8] Velmeden D., Söhne J., Schuch A., Zeid S., Schulz A., Troebs S., Müller F., Heidorn M.W., Buch G., Belanger N. (2025). Role of Heart Rate Recovery in Chronic Heart Failure: Results From the MyoVasc Study. J. Am. Heart Assoc..

[9] Toledo C., Andrade D.C., Lucero C., Arce-Alvarez A., Díaz H.S., Aliaga V., Schultz H.D., Marcus N.J., Manríquez M., Faúndez M. (2017). Cardiac diastolic and autonomic dysfunction are aggravated by central chemoreflex activation in heart failure with preserved ejection fraction rats. J. Physiol..

[10] Bailey C.S., Wooster L.T., Buswell M., Patel S., Pappagianopoulos P.P., Bakken K., White C., Tanguay M., Blodgett J.B., Baggish A.L. (2018). Post-Exercise Oxygen Uptake Recovery Delay. JACC Heart Fail..

[11] Cozgarea A., Cozma D., Teodoru M., Lazăr-Höcher A.-I., Cirin L., Faur-Grigori A.-A., Lazăr M.-A., Crișan S., Gaiță D., Luca C.-T. (2024). Heart Rate Recovery: Up to Date in Heart Failure—A Literature Review. J. Clin. Med..

[12] Dache A., Văcărescu C., Teodoru M., Negrea M.O., Lazăr-Höcher A., Cirin L., Faur-Grigori A.A., Suciu B.S., Gaiță D., Luca C.-T. (2025). Heart Rate Recovery Index as a Novel Marker in Heart Failure Assessment: A Comparative Analysis of Heart Rate Deceleration During Exercise Testing. Int. J. Gen. Med..

[13] McDonagh T.A., Metra M., Adamo M., Gardner R.S., Baumbach A., Böhm M., Burri H., Butler J., Čelutkienė J., Chioncel O. (2023). 2023 Focused Update of the 2021 ESC Guidelines for the diagnosis and treatment of acute and chronic heart failure. Eur. Heart J..

[14] Nagueh S.F., Smiseth O.A., Appleton C.P., Byrd B.F., Dokainish H., Edvardsen T., Flachskampf F.A., Gillebert T.C., Klein A.L., Lancellotti P. (2016). Recommendations for the Evaluation of Left Ventricular Diastolic Function by Echocardiography: An Update from the American Society of Echocardiography and the European Association of Cardiovascular Imaging. J. Am. Soc. Echocardiogr..

[15] Praz F., A Borger M., Lanz J., Marin-Cuartas M., Abreu A., Adamo M., Marsan N.A., Barili F., Bonaros N., Cosyns B. (2025). 2025 ESC/EACTS Guidelines for the management of valvular heart disease. Eur. Heart J..

[16] Araz K., Fioretti F., Ladak S., Obaidan M., Butler J., Hameed A. (2025). Phenotype Characterization of Heart Failure with Preserved Ejection Fraction in Medical Device and Surgical Trials. ESC Heart Fail..

[17] Suciu B.-S., Turi V.R., Crisan S., Luca C.T., Lazar D.-C., Faur-Grigori A.A., Petrescu M., Dache A., Cioca F., Văcărescu C. (2025). Heart Failure with Preserved Ejection Fraction (HFpEF), Pulse Wave Velocity, and Heart Rate Recovery Interconnections—A Brief Literature Review. J. Clin. Med..

[18] Bernardi M., Golino M., Spadafora L., Sarto G., Forte M., Simeone B., Rocco E., Valenti V., Schiavon S., Esposito A. (2025). Current Challenges for the Diagnosis of HFpEF and Possible Simplification of the Diagnostic Approach. J. Cardiovasc. Pharmacol..

[19] Hwang I.-C., Cho G.-Y., Choi H.-M., Yoon Y.E., Park J.J., Park J.-B., Park J.-H., Lee S.-P., Kim H.-K., Kim Y.-J. (2021). H2FPEF Score Reflects the Left Atrial Strain and Predicts Prognosis in Patients with Heart Failure with Preserved Ejection Fraction. J. Card. Fail..

[20] Jorge A.J.L., Rosa M.L.G., Martins W.d.A., Leite A., Correia D.M.d.S., Saad M.A.N., Villacorta H., Chermont S., Gismondi R.A., Almeida B.M. (2020). Prognosis of Heart Failure with Preserved Ejection Fraction in Primary Care by the H2FPEF Score. Int. J. Cardiovasc. Sci..

[21] Goette A., Corradi D., Dobrev D., Aguinaga L., Cabrera J.-A., Chugh S.S., de Groot J.R., Soulat-Dufour L., Fenelon G., Hatem S.N. (2024). Atrial cardiomyopathy revisited—Evolution of a concept: A clinical consensus statement of the European Heart Rhythm Association (EHRA) of the ESC, the Heart Rhythm Society (HRS), the Asian Pacific Heart Rhythm Society (APHRS), and the Latin American Heart Rhythm Society (LAHRS). Europace.

[22] Cheng Y., Zhang L., Li L., Fei M., Wang Y., Zhang P. (2026). Clinical utility of the four-dimensional automatic left atrial quantification technique in evaluating left atrial volume and function in patients with heart failure with preserved ejection fraction. Quant. Imaging Med. Surg..

[23] Popescu B.A., Beladan C.C., Nagueh S.F., Smiseth O.A. (2022). How to assess left ventricular filling pressures by echocardiography in clinical practice. Eur. Heart J. Cardiovasc. Imaging.

[24] Würzburger L., van der Stouwe J.G., Ghidoni C., Wiech P., Moser G., Petrasch G., Schweiger V., Bohm P., Rossi V.A., Templin C. (2024). Blood pressure behavior during exercise in patients with diastolic dysfunction and a hypertensive response to exercise. J. Clin. Hypertens..

[25] Mashayekhi B., Mohseni-Badalabadi R., Hosseinsabet A., Ahmadian T. (2023). Correlation between Heart rate recovery and Left Atrial phasic functions evaluated by 2D speckle-tracking Echocardiography after Acute Myocardial infarction. BMC Cardiovasc. Disord..

[26] Barakat M.F., Chehab O., Kaura A., Sunderland N., Hayat S., Dhillon P.S., Gall N., Monaghan M.J., Amin-Youssef G., Mayet J. (2020). Tissue Doppler-Derived Left Ventricular Systolic Velocity Is Associated with Lethal Arrhythmias in Cardiac Device Recipients Irrespective of Left Ventricular Ejection Fraction. J. Am. Soc. Echocardiogr..

[27] Chawla S., Sato R., Duggal A., Alwakeel M., Hasegawa D., Alayan D., Collier P., Sanfilippo F., Lanspa M., Dugar S. (2023). Correlation between tissue Doppler-derived left ventricular systolic velocity (S’) and left ventricle ejection fraction in sepsis and septic shock: A retrospective cohort study. J. Intensive Care.

[28] Cirin L., Crișan S., Luca C.-T., Buzaș R., Lighezan D.F., Văcărescu C., Cozgarea A., Tudoran C., Cozma D. (2024). Mitral Annular Plane Systolic Excursion (MAPSE): A Review of a Simple and Forgotten Parameter for Assessing Left Ventricle Function. J. Clin. Med..

[29] Falconer D., Fröjdh F., Wyeth N., Brieger D., Captur G., Kozor R., Ugander M. (2026). The diagnostic and prognostic utility of mitral annular plane systolic excursion (MAPSE): A systematic review and Meta-Analysis. J. Am. Heart Assoc..

[30] Tanaka S., Miyamoto T., Mori Y., Harada T., Tasaki H. (2021). Heart rate recovery is useful for evaluating the recovery of exercise tolerance in patients with heart failure and atrial fibrillation. Heart Vessel..

